# Supplementary material for: Nuclear export and translation of circular repeat-containing intronic RNA in C9ORF72-ALS/FTD
Source: Nat Commun. 2021 Aug 13;12:4908. doi: 10.1038/s41467-021-25082-9 (PMC8363653; doi:10.1038/s41467-021-25082-9)
Supplement: Supplementary file 1 — Supplementary information [file 41467_2021_25082_MOESM1_ESM.pdf]

Supplementary Information

Nuclear export and translation of circular repeat-containing intronic RNA in C9ORF72-ALS/FTD

Shaopeng Wang[1,2*], Malgorzata J. Latallo[3,4*], Zhe Zhang[1,2], Bo Huang[1,5], Dmitriy G. Bobrovnikov[3], Daoyuan Dong[1,2], Nathan M. Livingston[3,4], Wilson Tjoeng[1,2], Lindsey R. Hayes[2,6], Jeffrey D. Rothstein[2,6], Lyle W. Ostrow[6], Bin Wu[3,4,7#], Shuying Sun[1,2,8#]

[1]Department of Pathology, Johns Hopkins University School of Medicine, Baltimore, MD 21205
[2]Brain Science Institute, Johns Hopkins University School of Medicine, Baltimore, MD 21205
[3]Department of Biophysics and Biophysical Chemistry, Johns Hopkins University School of Medicine, Baltimore, MD 21205
[4]Center for Cell Dynamics, Johns Hopkins University School of Medicine, Baltimore, USA
[5]Sol Goldman Pancreatic Cancer Research Center, Johns Hopkins University School of Medicine, Baltimore, MD, 21205
[6]Department of Neurology, Johns Hopkins University School of Medicine, Baltimore, MD 21205
[7]Solomon H. Snyder Department of Neuroscience, Johns Hopkins University School of Medicine, Baltimore, USA
[8]Department of Physiology, Johns Hopkins University School of Medicine, Baltimore, MD 21205
[*]These authors contributed equally to this work

#Corresponding authors:

Shuying Sun (shuying.sun@jhmi.edu); Bin Wu (bwu20@jhmi.edu)

Lead contact: Shuying Sun

Supplementary Figures

Supplementary Figure 1

Characterization of C9ORF72 splicing reporter cells

Supplementary Figure 2

Nanopore RNA sequencing to characterize the RNA molecules generated from the C9ORF72-$(GGGGCC)_{70}$ splicing reporter

Supplementary Figure 3

Nanopore RNA sequencing to characterize the RNA molecules generated from the C9ORF72-$(GGGGCC)_{70}$ splicing reporter in HEK293T cells

Supplementary Figure 4

Characterization of RNA granules and single RNA molecules in C9ORF72 splicing reporter cells

Supplementary Figure 5

smFISH of endogenous *C9ORF72* intron 1 and mRNA

Supplementary Figure 6

The linear RNAs containing the MBS or $GGGGCC^{exp}$ were efficiently degraded by RNase R treatment

Supplementary Figure 7

The repeat-containing intron is exported in the circular form

Supplementary Figure 8

Circular intron of *C9ORF72* in patient samples

Supplementary Figure 9

G-rich repeats stabilize the spliced intron and mediate the nuclear export

Supplementary Figure 10

G-rich repeats stabilize spliced introns in nucleus

Supplementary Figure 11

Translation of circular repeat-containing intron and NXF1-NXT1-mediated export

Supplementary Figure 12

Genome-wide circular introns have higher G-content and are more structured

Supplementary tables

Supplementary table 1

Patient cell lines used in the study

Supplementary table 2

Patient postmortem tissues used in the study

Supplementary table 3

Primers for qPCR and PCR

Supplementary table 4

Primers for MiSeq library preparation

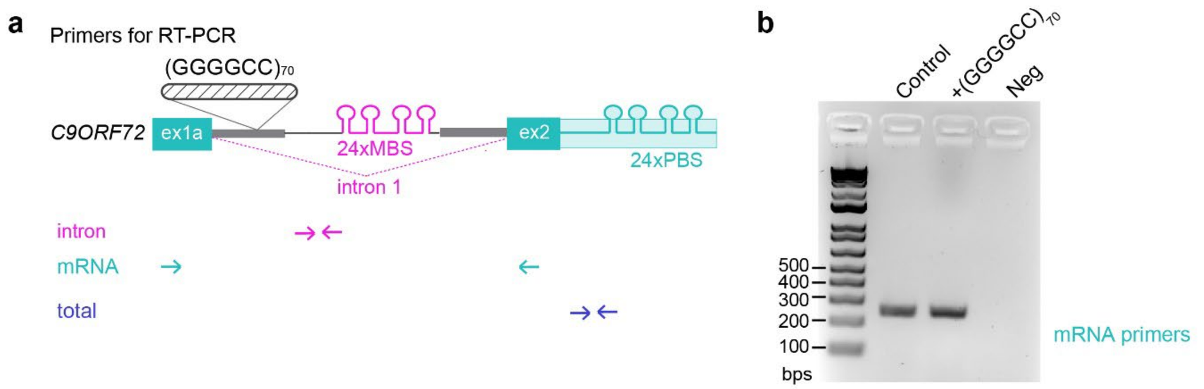

Supplementary Fig. 1 | Characterization of C9ORF72 splicing reporter cells. a, Schematic of C9ORF72 splicing reporter construct and the positions of primers amplifying different RNA species. The "intron" primers (magenta) targeting the MBS region were used to measure intron RNA level in Fig. S3e. The "mRNA" primers (cyan) targeting exon 1a and exon 2 were used in (b). The "total" primers (blue) targeting the region before PBS were used to quantify the total transgene level in Fig. S3d. **b**, PCR amplification of spliced mRNA from the reporters with and without the expanded repeats in the intron. The band showed expected size of spliced mRNA in both reporter cell lines. Total three independent biological replicates were examined with similar results.

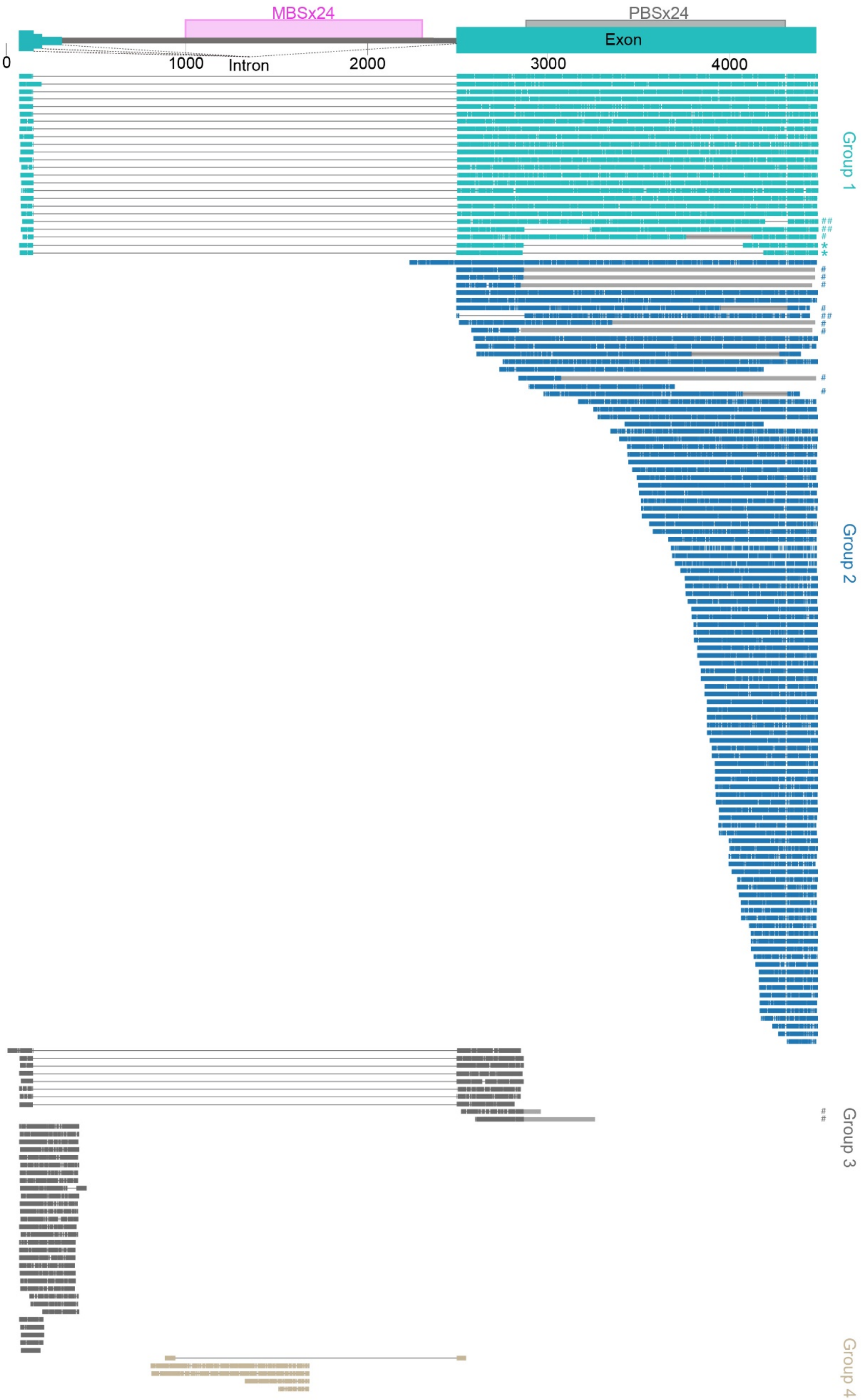
MBSx24
PBSx24
Exon
Intron
0
1000
2000
3000
4000
Group 1
Group 2
Group 3
Group 4

Supplementary Fig. 2 | Nanopore RNA sequencing to characterize the RNA molecules generated from the C9ORF72-(GGGGCC)$_{70}$ splicing reporter. Alignment of Nanopore long-read sequencing of poly(A) enriched mRNAs to ascertain different RNA isoforms from the reporter transgene. The primary reads alignment was performed by the nanopore direct RNA sequencing splice alignment mode (Methods). From the 1.53 million raw sequences, 171 reads can be aligned with high confidence. Due to the repetitive sequences in 24×PBS, some reads were mapped incompletely and showed non-existing gaps in the alignment. We used the EMBOSS Needle tool as an alternative method, which partially improved the alignments. # Grey-shaded regions can be mapped to the transgene using the EMBOSS Needle tool. ## Sequences that have correct length (no gaps) but could not be fully aligned with either mapping method. *Sequences with shorter length, indicating the gap is not mis-alignment. Group 1: Twenty-four mRNA sequences span from the 5' (Ex1a, two alternative 5'ss) to the expected poly(A) sites. There were two molecules missing significant chunk of PP7 sites (*marked), invisible in the imaging experiment. Group 2: 5' truncated transcripts with expected poly(A) sites (read in the 3'→5' direction), a common phenomenon inherent in Nanopore sequencing. Group 3: a small group of previously unidentified short reads starting from Exon 1a and ending either inside intron 1 (before the repeats) or in exon 2 (before the PP7 binding sites). These transcripts contained neither MS2, nor PP7 binding sites, therefore invisible in the imaging experiment. Group 4: Molecules with a small segment of MS2 and some random sequences that cannot be mapped correctly to our construct, which might be due to errors during the stable integration or sequencing error.

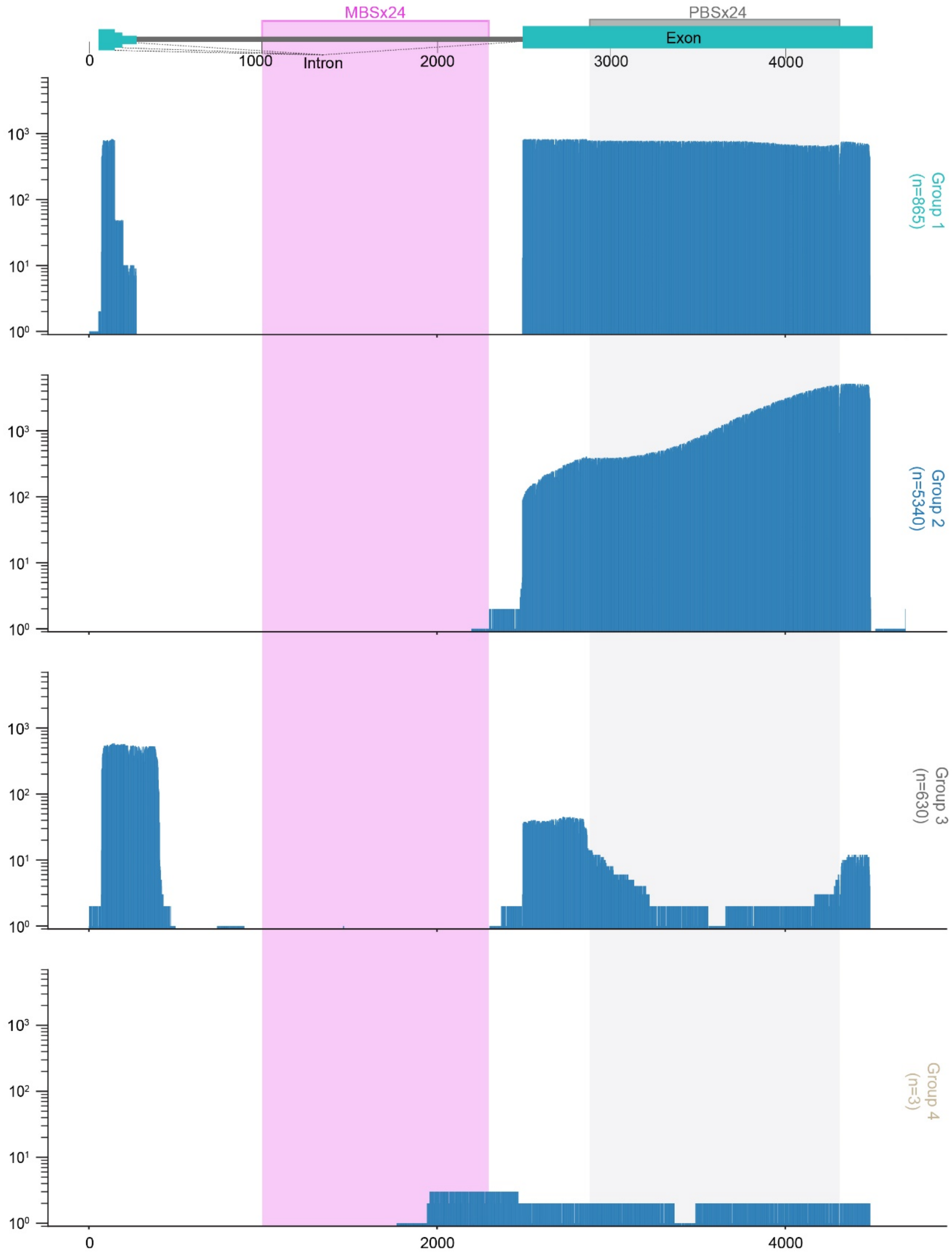
MBSx24
PBSx24
Exon
Intron
0
1000
2000
3000
4000
Group 1
(n=865)
Group 2
(n=5340)
Group 3
(n=630)
Group 4
(n=3)
10^3
10^2
10^1
10^0

Supplementary Fig. 3 | Nanopore RNA sequencing to characterize the RNA molecules generated from the C9ORF72-(GGGGCC)$_{70}$ splicing reporter in HEK293T cells. Alignment of Nanopore long-read sequencing of poly(A) enriched mRNAs to ascertain different RNA isoforms from the reporter transfected into HEK 293T cells. The primary reads alignment was performed by the nanopore direct RNA sequencing splice alignment mode (Methods). From the 0.84 million raw sequences, 6838 reads can be aligned with high confidence. Each of the panels represents the identified read coverage in logarithmic scale. The pink shaded area represents MBS, the gray shaded area represents PBS sequence region. Group 1: Expected mRNA sequences which span from the 5' (Ex1a, three alternative 5'ss) to the expected poly(A) sites. Group 2: 5' truncated transcripts with expected poly(A) sites (read in the 3'→5' direction), a common phenomenon inherent in Nanopore sequencing. Group 3: a group of previously unidentified short reads starting from Exon 1a and ending either inside intron 1 (before the repeats) or in exon 2. These transcripts contained neither MS2 nor PP7 binding sites, therefore invisible in the imaging experiment. Group 4: Molecules with a small segment of MS2.

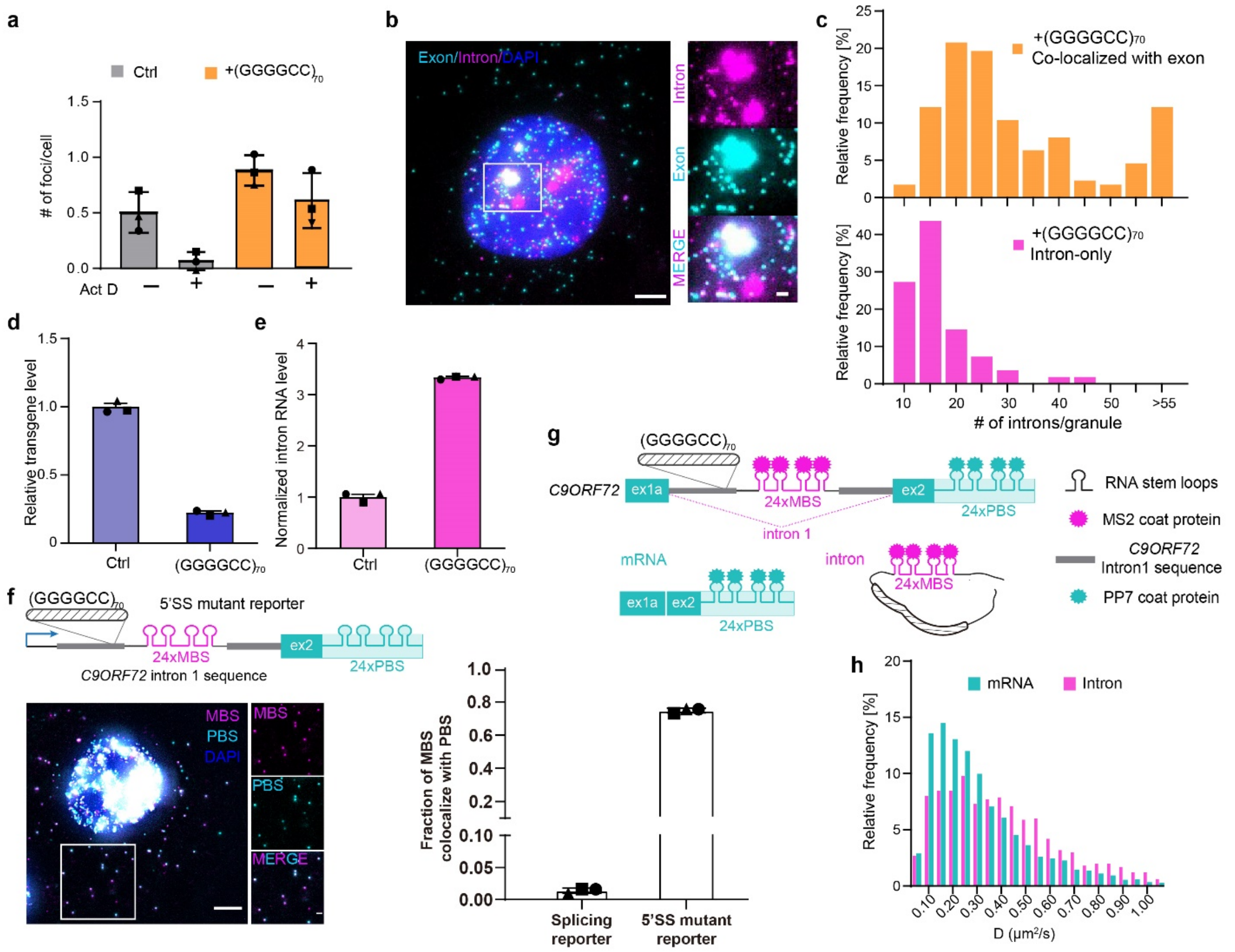

Supplementary Fig. 4 | Characterization of RNA granules and single RNA molecules in C9ORF72 splicing reporter cells. a, Number of nuclear RNA foci per cell before or after 20-minute treatment of 1µg/mL Actinomycin D, a transcription inhibitor. RNA foci in control cells were transcription sites. Data are mean ± SD from three biological replicates (n=60, 43, 93 cells in Ctrl without Act D; n=93, 95, 49 cells in (GGGGCC)$_{70}$ without Act D; n=88, 82, 41 cells in Ctrl with Act D; n=72, 43, 73 cells in (GGGGCC)$_{70}$ with Act D). **b**, Representative images of two-color smFISH in +(GGGGCC)$_{70}$ reporter cells containing intron-only RNA granules. Magenta: intron; cyan: exon; blue: DAPI. Scale bar: 5 µm. The boxes on the left were zoomed and shown on the right, scale bar: 1 µm. Total 45 out of 188 cells have intron only granules. **c**, Size distribution of intron-exon colocalized granules (top) and intron-only granules (bottom). The number of intron RNA molecules per foci was calculated by dividing the integrated intensity value of foci over mean single intron intensity in the experimental set, from 237 cells in three biological replicates. **d**, The relative transgene expression levels in control and +(GGGGCC)$_{70}$ reporter cells were measured by qRT-PCR using primers for total RNA as shown in Fig. S1a. Data are mean ± SEM from three biological replicates. **e**, The intron levels were measured by qRT-PCR using primers for intron RNA (Fig. S1a) and normalized to the corresponding total transgene levels (d) to show

the relative steady state intron levels in control and +$(GGGGCC)_{70}$ reporter cells. Data are mean ± SEM from three biological replicates. **f**, smFISH of the 5'ss mutant reporter. Magenta: MBS; cyan: PBS; blue: DAPI. Scale bar: 5 μm. Zoom-in scale bar: 1 μm. Quantification of co-localized MBS-PBS molecules in splicing reporter and 5'ss mutant reporter was shown on the left. (6108 cytoplasmic introns for splicing reporter and 2633 for 5'ss mutant reporter, from three biological replicates). **g**, Schematic of the C9ORF72 splicing reporter construct, which can be used for live cell imaging. The 24×MBS tagged RNA was fluorescently labeled by MS2 coat proteins (MCP), and the 24×PBS tagged RNA was labeled by PP7 coat proteins (PCP). **h,** The histogram of calculated diffusion coefficients of single exon and intron RNA molecules in cytoplasm. The histogram represents the results of RNA tracking from 5 cells (1003 traces of exon and 681 traces of intron RNA). Source data are provided as a Source Data File.

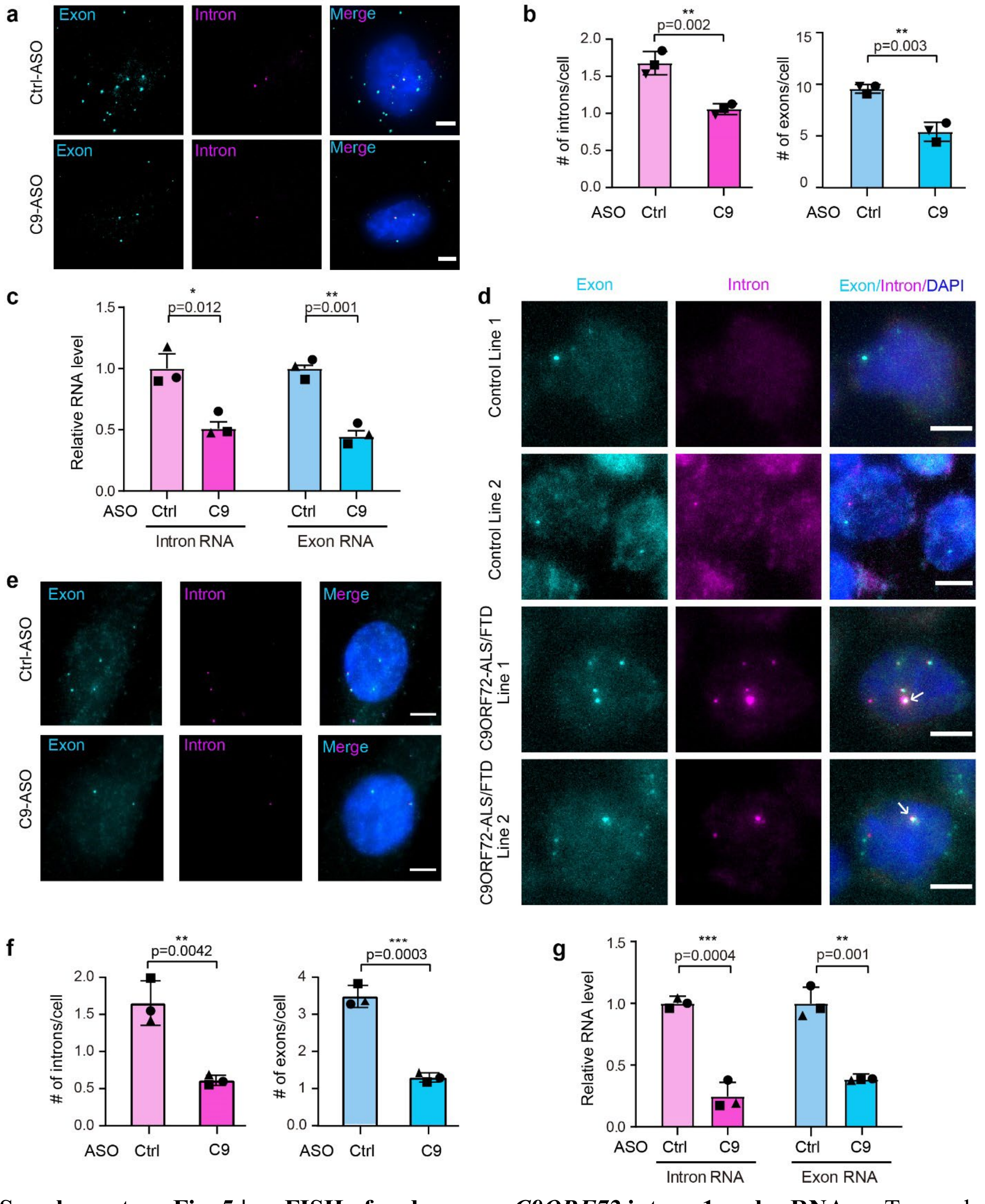

Supplementary Fig. 5 | smFISH of endogenous *C9ORF72* intron 1 and mRNA. a, Two-color smFISH of endogenous *C9ORF72* RNAs in U-2 OS cells transfected with non-targeting control or C9-targeting ASO. A set of 76 probes targets intron1 (magenta) and a 77-probe set targets all the exons (cyan). Scale bar: 5 µm. **b**, Quantification of total intron and exon RNA molecules per

cell from smFISH. Three shapes represented three biological replicates (Ctrl ASO: 95, 92, 62 cells quantified; C9 ASO: 74, 48, 112 cells quantified). The mean of each replicate was used to calculate the average (horizontal bar) and SD (error bars) in each group, as well as for statistic comparison between groups. ***P*<0.01, two-tailed *t* test. **c**. Endogenous *C9ORF72* intron1 and mRNA levels were measured by qRT-PCR in U-2 OS cells transfected with control or C9-targeting ASO. Data are mean ± SEM from three biological replicates. **P*<0.05, ***P*<0.01, two-tailed *t* test. **d**, Two-color smFISH of endogenous *C9ORF72* RNAs in lymphoblast cells. Scale bar: 5 µm. Arrow: nuclear RNA granules containing both intron and exon, which were only present in patient cells. Three independent experiments were carried out with similar results. **e**. smFISH of endogenous *C9ORF72* RNAs in patient fibroblast cells transfected with non-targeting control or C9-targeting ASO as in **a**. Scale bar: 5 µm. **f**. Quantification of total intron and exon RNA molecules per cell from the smFISH. Three shapes represented three biological replicates (Ctrl ASO: 57, 63, 66 cells quantified; C9 ASO: 73, 68, 30 cells quantified). **P<0.01, ****P*<0.001, two-tailed t test. **g**. Endogenous *C9ORF72* intron1 and mRNA levels were measured by qRT-PCR. Data are mean ± SEM from three biological replicates. **P<0.01, ****P*<0.001, two-tailed t test. Source data are provided as a Source Data File.

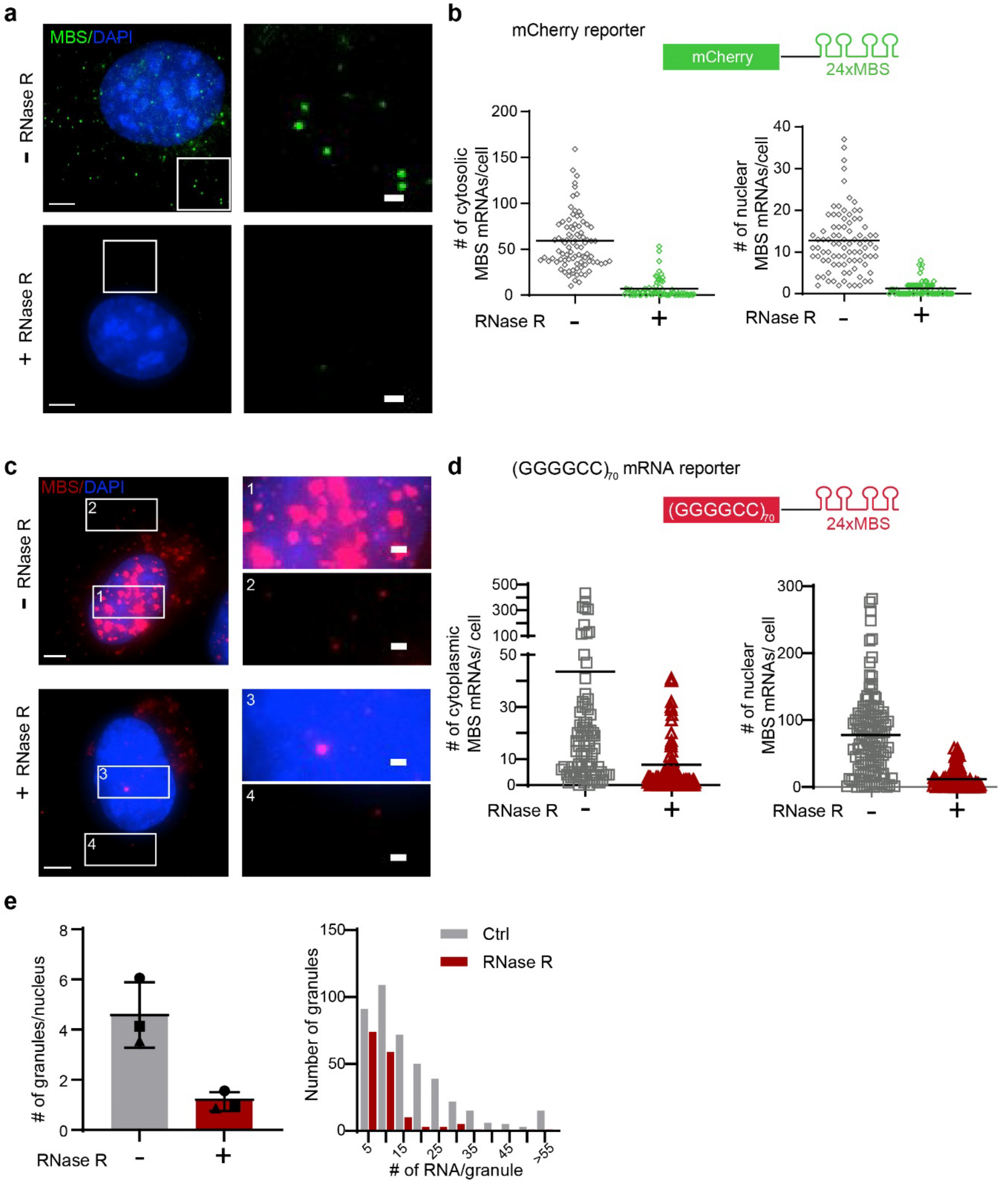

Supplementary Fig. 6 | The linear RNAs containing the MBS or GGGGCC^{exp} were efficiently degraded by RNase R treatment. a, Representative images of smFISH in mCherry-24×MBS mRNA reporter cells treated with RNase R (bottom) or just buffer (top). Green: mCherry (MBS); blue: DAPI. Scale bar: 5um, and 1 µm for zoom in. **b**, Quantification of cytosolic (left) and nuclear (right) mCherry-24×MBS mRNA per cell with the treatment of RNase R or buffer only. Each

symbol represented a single cell. The horizontal bar represents the mean value from a single experiment (89 cells for control and 69 cells for RNase R treatment). **c**, Representative images of smFISH in $(GGGGCC)_{70}$-24×MBS mRNA reporter cells treated with RNase R (bottom) or just buffer (top). Red: $(GGGGCC)_{70}$ (MBS); blue: DAPI. Scale bar: 5um, and 1 μm for zoom in. **d**, Quantification of cytosolic (left) and nuclear (right) $(GGGGCC)_{70}$-24×MBS mRNA per cell with or without RNase R treatment. Each symbol represented a single cell. The horizontal bar represents the mean value from a single experiment (126 cells for control and 98 cells for RNase R treatment). **e**, (Left) Number of RNA granules per cell with the treatment of RNase R or buffer only. Data are mean ± SD from three technical replicates (126 cells for control and 98 cells for RNase R treatment). (Right) Size distribution of RNA granules with or without RNase R treatment. The number of intron RNA molecules per granule were calculated by dividing the integrated intensity value of each granule over mean single RNA value from 126 control cells. Source data are provided as a Source Data File.

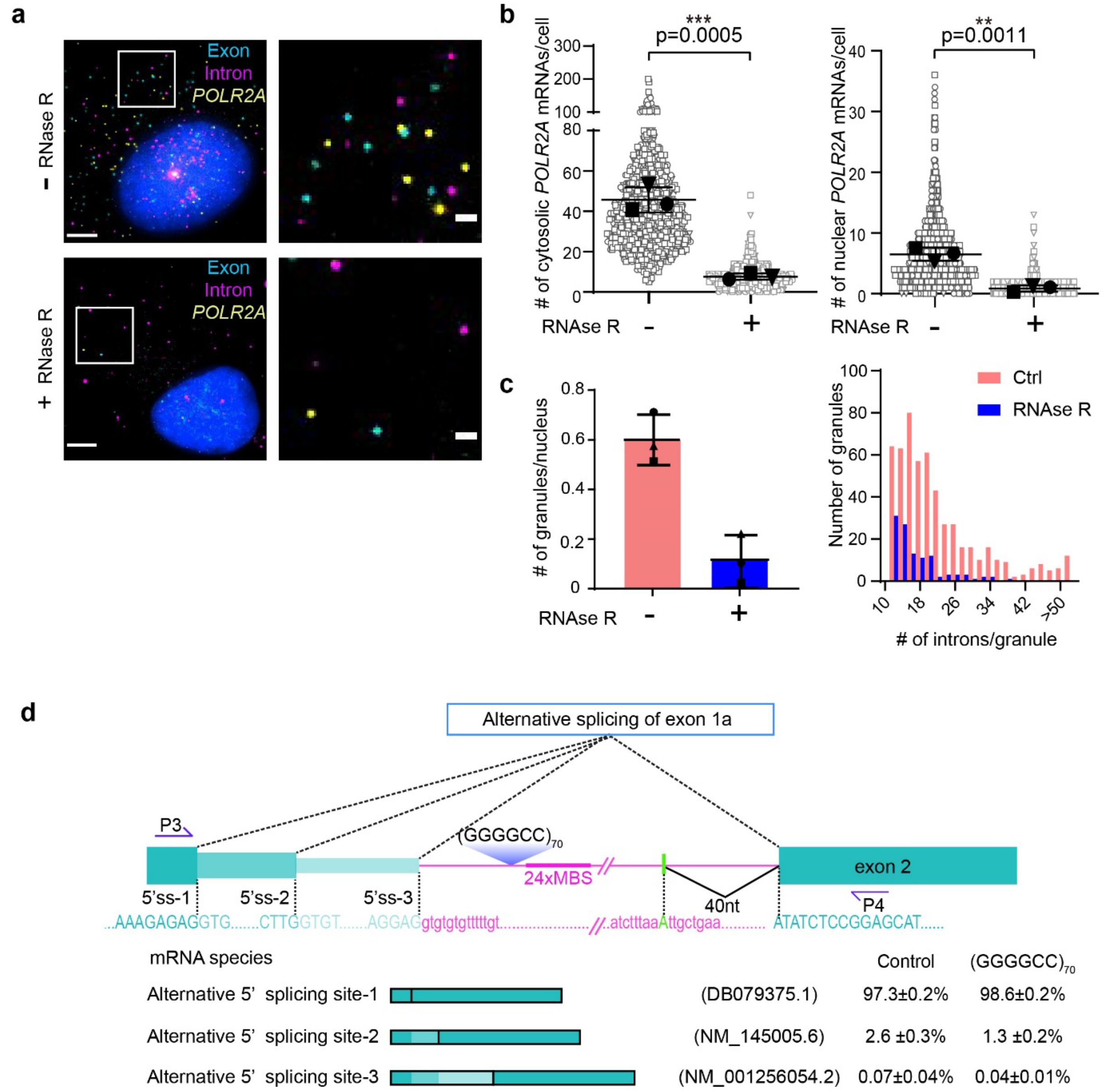

Supplementary Fig. 7 | The repeat-containing intron is exported in the circular form. a, Representative images of three-color smFISH in +(GGGGCC)$_{70}$ splicing reporter cells treated with RNase R (bottom) or just buffer (top) (as in Fig. 3). Magenta: intron (MBS); cyan: exon (PBS); yellow: *POLR2A*; blue: DAPI. Scale bar: 5um, and 1 μm for zoom in. **b**, Quantification of cytosolic (left) and nuclear (right) *POLR2A* mRNA per cell with the treatment of RNase R or buffer only. Each symbol represented a single cell and the three shapes represented three technical replicates. The mean of each replicate (larger black shapes) was used to calculate the average (horizontal bar) and SD (error bars) in each group, as well as for statistic comparison between groups. ** $P<0.01$, *** $P<0.001$, two-tailed t test. **c**, (Left) Number of repeat RNA granules per cell with the treatment

of RNase R or buffer only. Data are mean ± SD from three technical replicates. (Right) Size distribution of RNA granules with or without RNase R treatment. The number of intron RNA molecules per granule were calculated by dividing the integrated intensity value of each granule over mean single RNA value in experimental set from three technical replicates (The cell number in (**b**) and (**c**) from each replicate is: 287, 289, 263 for RNase R treatment, and 222, 369, 342 for control). **d**, Deep sequencing identified alternative 5' splice site (5'ss) of *C9ORF72* exon 1a. The libraries were prepared using the indicated primers (P3-P4), and the whole fragments were sequenced by 1×300nt MiSeq. The reads spanning the exon-exon junctions were aligned. The most upstream 5'ss is predominantly used. Data are mean ± SD of three technical replicates. Source data are provided as a Source Data File.

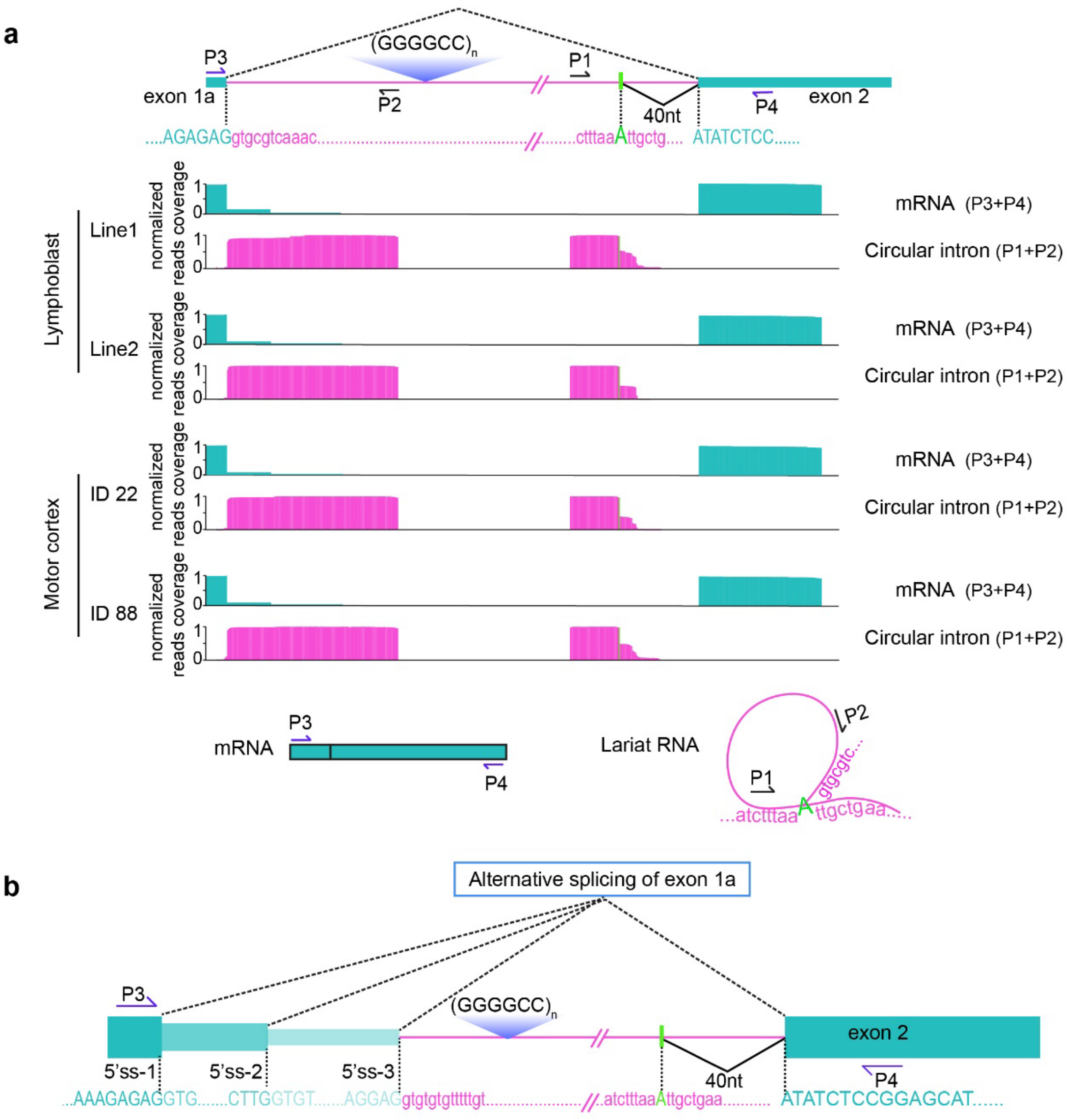

mRNA species	Lymphoblast Control	Lymphoblast (GGGGCC)n	Motor cortex Control	Motor cortex (GGGGCC)n
Alternative 5' splicing site-1 (DB079375.1)	90.7±0.7%	90.2±4.1%	96.6±0.4%	93.7±1.1%
Alternative 5' splicing site-2 (NM_145005.6)	8.0±0.8%	8.7±3.8%	2.9±0.3%	5.6±1.7%
Alternative 5' splicing site-3 (NM_001256054.2)	1.35±0.12%	1.1±0.28%	0.48±0.66%	0.7±0.56%

Supplementary Fig. 8 | Circular intron of *C9ORF72* in patient samples. a, Deep sequencing identified circular boundaries of endogenous *C9ORF72* intron 1 RNA, which is similar to the reporter. The major splicing junction in mRNAs and branch site in circular RNAs were annotated. The libraries were prepared using the indicated primers (P1-P2 for intron; P3-P4 for mRNA), and the whole fragments were sequenced by 1×300nt MiSeq. The reads spanning the exon-exon junctions in mRNAs were aligned, shown in cyan. The reads spanning the junction between branch

site and 5’-end of the intron in circular RNAs were aligned and shown in magenta. The most upstream 5’ss is the predominant splice site linked to the branch site. **b**, Deep sequencing identified alternative 5’ splice site (5’ss) of endogenous *C9ORF72* exon 1a. The libraries were prepared using the indicated primers (P3-P4), and the whole fragments were sequenced by 1×300nt MiSeq. The reads spanning the exon-exon junction were aligned. The most upstream 5’ss is predominantly used in the endogenous *C9ORF72* mRNA. Data are mean ± SD of two biological replicates. Source data are provided as a Source Data File.

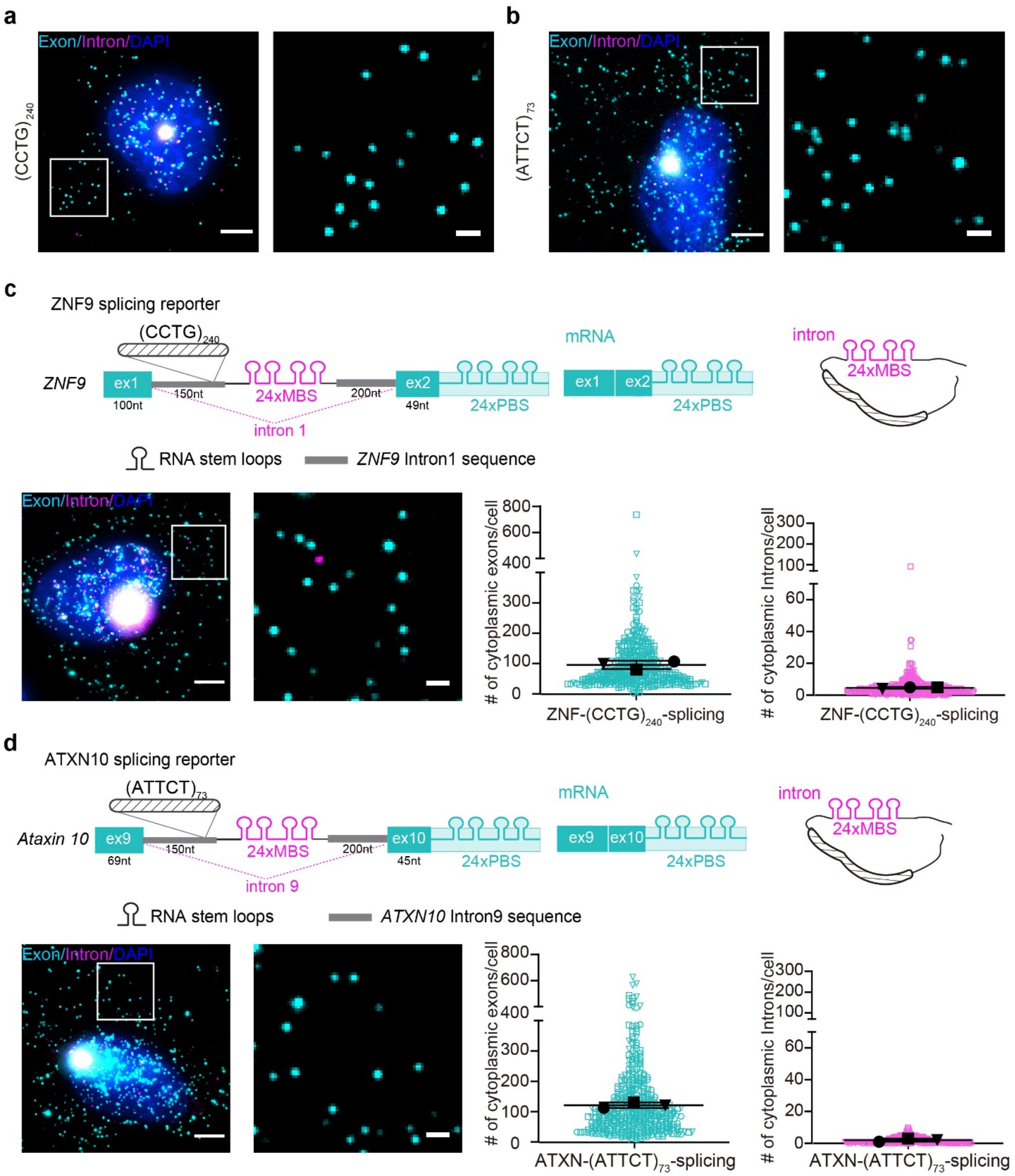

Supplementary Fig. 9 | G-rich repeats stabilize the spliced intron and mediate the nuclear export. a,**b**, Representative two-color smFISH of C9ORF72 splicing reporter cells containing intronic $(CCTG)_{240}$ (**a**) and $(ATTCT)_{73}$ (**b**) repeats (as in Fig. 5). Magenta: intron (MBS); cyan: exon (PBS); blue: DAPI. Scale bar: 5 μm and 1 μm for zoom-in respectively. **c**, ZNF9 splicing reporter with $(CCTG)_{240}$ in the intron. (Top) reporter diagram. MBS/PBS: MS2/PP7 binding sites; ex1, ex2: exon 1 and 2 of *ZNF9*. $CCTG^{exp}$ is located in the intron 1 of the *ZNF* gene. (Bottom left)

Representative smFISH image. Magenta: intron (MBS); cyan: exon (PBS); blue: DAPI. Scale bar: 5 µm, and 1 µm for zoom-in. (Bottom right) Quantification of cytoplasmic exon (cyan) and intron (magenta) numbers per cell. Each symbol represented a single cell and the three shapes represented three biological replicates (n=132, 156, 129 cells). The mean of each replicate (larger black shapes) was used to calculate the average (horizontal bar) and SD (error bars). **d**, ATXN10 splicing reporter with $(ATTCT)_{73}$ in the intron. (Top) reporter diagram. Ex9, ex10: exon 9 and 10 of *ATXN10*. $ATTCT^{exp}$ is located in the intron 9 of the *ATXN10* gene. (Bottom left) Representative smFISH image. Magenta: intron (MBS); cyan: exon (PBS); blue: DAPI. Scale bar: 5 µm, and 1 µm for zoom-in. (Bottom right) Quantification of cytoplasmic exon (cyan) and intron (magenta) numbers per cell. Each symbol represented a single cell and the three shapes represented three biological replicates (n=147, 139, 153 cells). The mean of each replicate (larger black shapes) was used to calculate the average (horizontal bar) and SD (error bars). Source data are provided as a Source Data File.

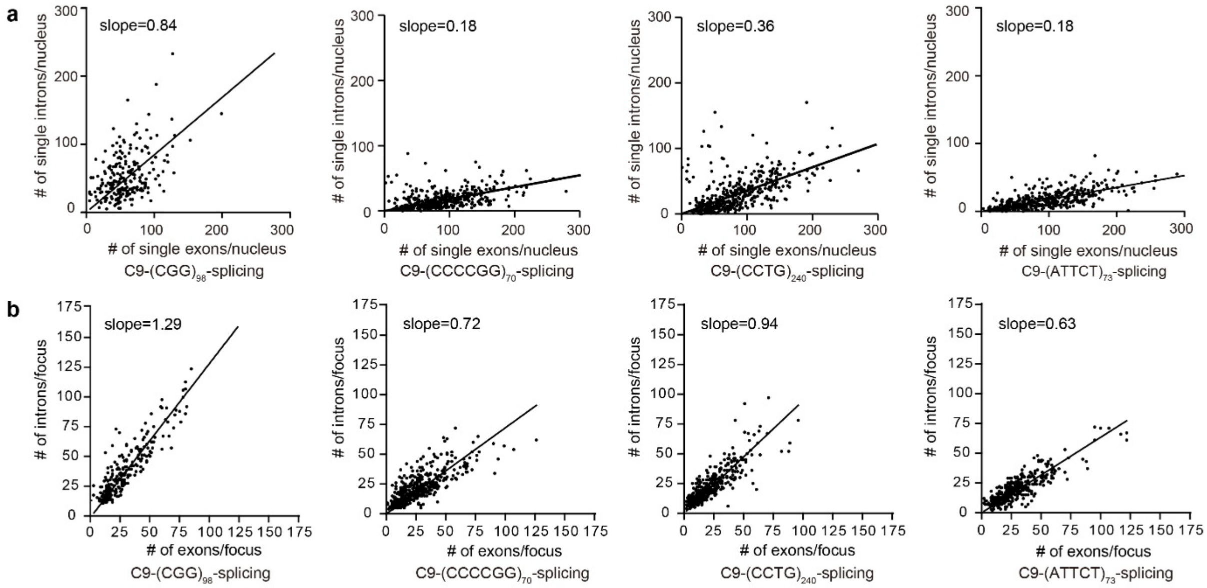

Supplementary Fig. 10 | G-rich repeats stabilize spliced introns in nucleus. a, Scatter plot of the numbers of single introns vs exons per nucleus in the C9ORF72 splicing reporters containing $(CGG)_{98}$, $(CCCCGG)_{70}$, $(CCTG)_{240}$ or $(ATTCT)_{73}$ repeat expansion (as in Fig. 4). Each dot represented a single nucleus. The lines were linear fit to the scatter plot. The slope reflects the ratio of intron *vs* exon molecules in each nucleus. **b**, Scatter plot of intron *vs* exon numbers in each RNA granule of the above four reporter cell lines. Each dot represented one granule. The lines were linear fit to the scatter plot. The slope reflects the ratio of intron *vs* exon molecules in each granule. The exact cell number quantified in both (**a**) and (**b**) from each replicate is: $(CGG)_{98}$: 75, 80, 73; $(CCCCGG)_{70}$: 162, 146, 103; $(CCTG)_{240}$: 147, 171, 119; $(ATTCT)_{73}$: 180,114,151. Source data are provided as a Source Data File.

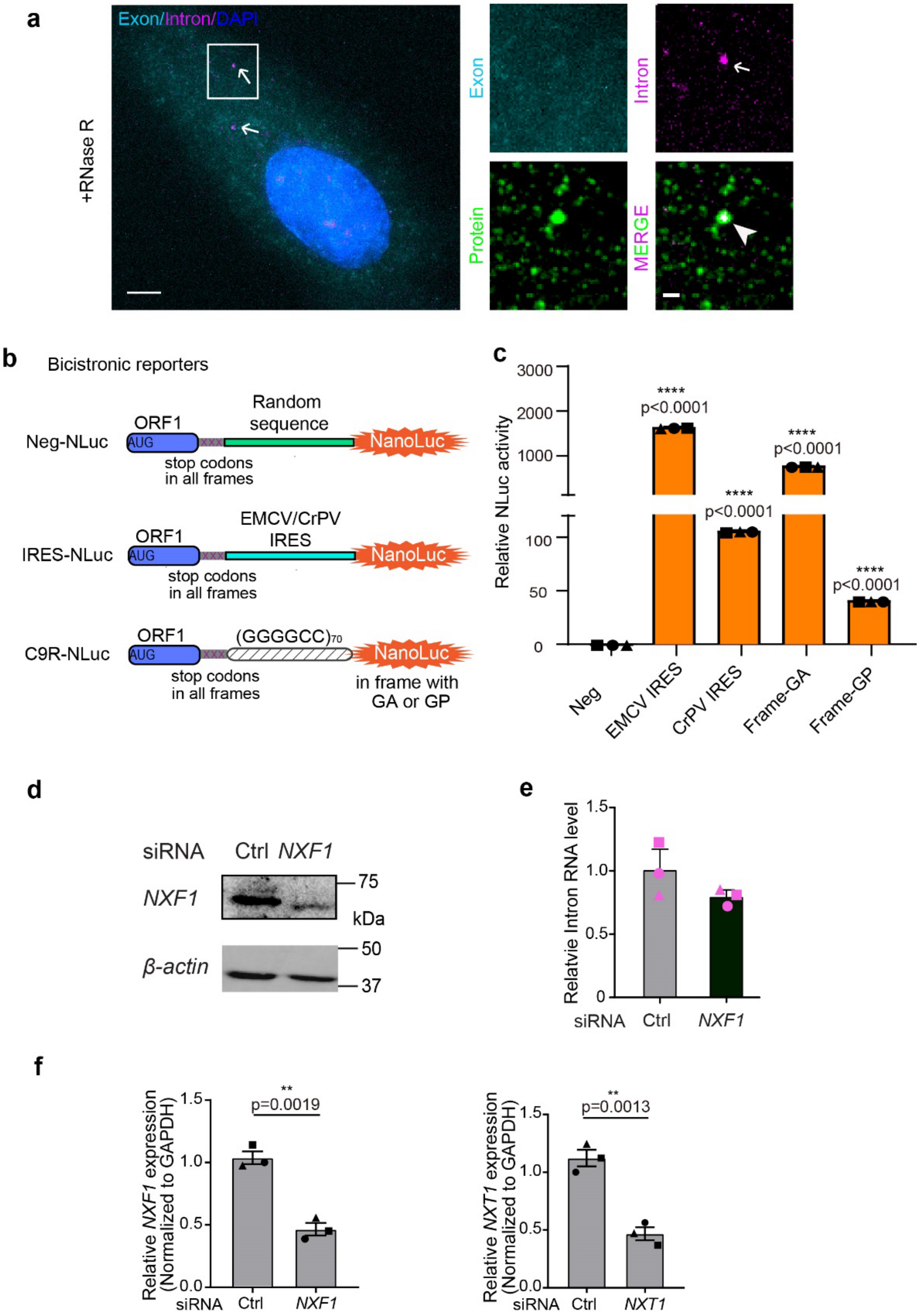

Supplementary Fig. 11 | Translation of circular repeat-containing intron and NXF1-NXT1-mediated export. a, Four-color smFISH-IF in +(GGGGCC)$_{70}$ translation reporter cells (as in Fig. 5) treated with RNase R. Cyan: exon (PBS); magenta: intron (MBS); green: protein; blue: DAPI. Scale bar: 5 µm. The boxes were zoomed and shown on the right, scale bar: 1 µm. Arrow: intron; arrowhead: translation site. Total 130 ctrl (without RNase R treatment) and 99 RNase R treated

cells were examined with similar results. **b**, Schematic of the bicistronic reporters to assess cap-independent translation of $(GGGGCC)_{70}$ repeats *in vitro*. The RNA was transcribed *in vitro* by T7 polymerase using the linearized constructs as template. **c**, NLuc activity was measured after *in vitro* translation reaction and normalized to the negative control. Data are presented as mean ± SD from three biological replicates. ****P<0.0001 by two-tailed t test. **d-e,** The $+(GGGGCC)_{70}$ splicing reporter cells (as in Fig. 1) were transfected with either non-targeting control or NXF1-targeting siRNA. **d**, Immunoblotting of NXF1 showed the knockdown efficiency. β-actin was blotted as internal control. Molecular weights are indicated in kilodaltons (kDa). Total three independent biological replicates were examined with similar results. **e**, Intron RNA of the reporter transgene was measured by qRT-PCR in control or NXF1 knockdown cells and normalized to *GAPDH* mRNA. Data are mean ± SEM from three biological replicates. **f**, NXT1 and NXF1 mRNA levels were measured by qRT-PCR in the siRNA transfected iPSNs, normalized to the non-targeting siRNA control. Data are mean ± SEM from three biological replicates. **P<0.01 by two-tailed t test. Source data are provided as a Source Data File.

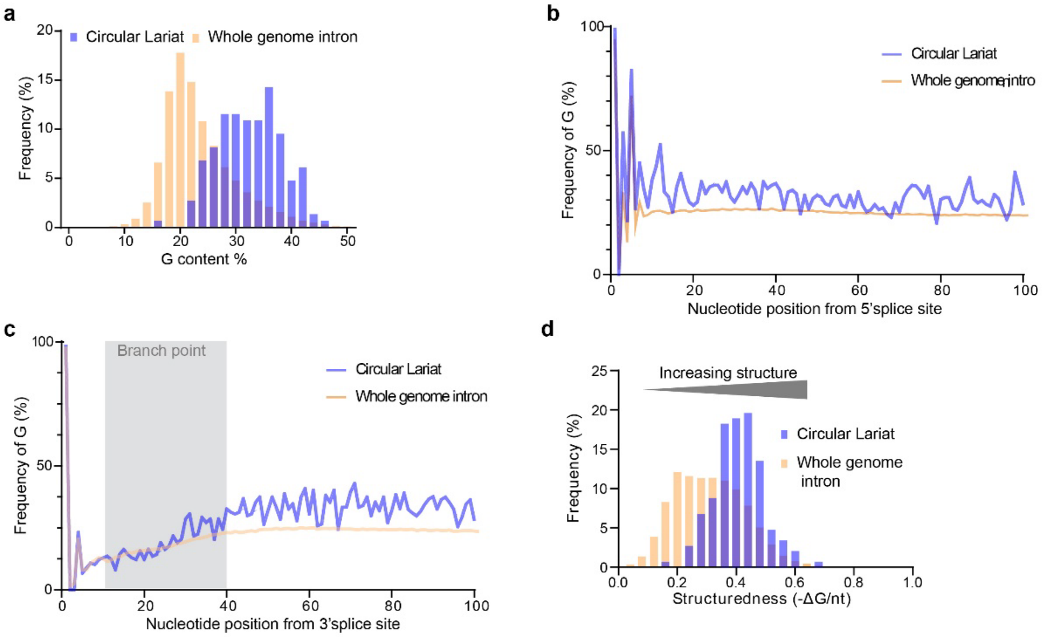

Supplementary Fig. 12 | Genome-wide circular introns have higher G-content and are more structured. **a**, Comparison of G content in the intron. Blue: circular intron in Hela cells (n=148, Talhouarne and Gall, 2018); Orange: all genomic introns longer than 55nt (n=205982). The circular lariats had a much higher G content (mean 32.5±5.7%) than that of the whole genomic introns (mean 23.5±6.5%). **b**, G percentage at each nucleotide position for the first 100 nucleotides of the intron starting from the 5' splicing site. **c**, G percentage at each nucleotide position for the last 100 nucleotides of the intron before the 3' splicing site. Blue: circular intron in HeLa cells (n=148, Talhouarne and Gall, 2018); Orange: all genomic introns (n=205982). A higher G percentage per nucleotide level for the circular lariat compared with genomic introns is observed, indicating the important role of high G content in proximity to the branch point. **d**, Comparison of predicted RNA structure (-ΔG/nt) of circular lariat introns from HeLa cells (n=148, blue)[29] and genome-wide introns between 100nt and 600nt (n= 64159, orange). The minimum free energy (ΔG) per nucleotide of the predicted RNA secondary structure was used as the proxy for intron structure complexity. Compared to the whole genome introns with a length between 100 and 600 nt (mean - ΔG/nt = 0.311), the circular lariats had lower free energy (mean - ΔG/nt = 0.408) indicating more structured. Altogether, these findings suggest that the high G content, particularly in proximity to the branch point, and structured elements in the intron play significant roles in debranching inhibition and circular intron stabilization. Source data are provided as a Source Data File.

Supplementary Tables

Supplementary Table 1. Patient cell lines used in the study.

Cell Line ID	Cell type	Line Name	ALS mutation	Gender
C9-ALS1	Lymphoblast	ND11411	C9ORF72 repeat expansion	Male
C9-ALS2	Lymphoblast	ND12455	C9ORF72 repeat expansion	Male
Control1	Lymphoblast	24312	NA	Female
Control2	Lymphoblast	38476	NA	Male
C9-ALS1	Fibroblast	C9-2	C9ORF72 repeat expansion	Male
C9-ALS2	Fibroblast	C9-5	C9ORF72 repeat expansion	Male
C9-ALS3	Fibroblast	C9-6	C9ORF72 repeat expansion	Male
Control1	Fibroblast	Ctrl-1	NA	Female
Control2	Fibroblast	Ctrl-2	NA	Female
C9-ALS1	iPSC	CS7VCZiALS	C9ORF72 repeat expansion	Male
C9-ALS2	iPSC	CS0BUUiALS	C9ORF72 repeat expansion	Female
C9-ALS3	iPSC	CS0NKCiALS	C9ORF72 repeat expansion	Female
Control1	iPSC	CS0594iCTR	NA	Female
Control2	iPSC	CS0702iCTR	NA	Male
Control3	iPSC	CS1ATZiCTR	NA	Male

Supplementary Table 2. Patient postmortem tissues used in the study.

Sample ID	CNS regions	Group	Gender	Age at Death	PMI(hrs)
22	Motor Cortex	C9ORF72 ALS	Male	66	6
88	Motor Cortex	C9ORF72 ALS	Male	59	NA
101	Motor Cortex	Control	Female	70	32
108	Motor Cortex	Control	Male	72	14

(PMI = post-mortem interval)

Supplementary Table 3. Primers for qPCR and PCR.

GAPDH mRNA	Forward	5'-GAGTCAACGGATTTGGTCGT-3'
	Reverse	5'-TTGATTTTGGAGGGATCTCG-3'
Reporter intron RNA (MS2)	Forward	5’-CCAATCAAACAGAAGCACCA-3’
	Reverse	5’-AGCCAGACATGCCGATATTC-3’
Reporter total RNA (FLUC)	Forward	5’-AGCTGCACAAAGCCATGAAG-3’
	Reverse	5’- TCTCGAAGTACTCGGCGTAG-3’
Reporter mRNA	Forward	5’-AGCTCTAGCAGGAAAGAG-3’
	Reverse	5’-TCTCGAAGTACTCGGCGTAG-3’
NXT1 mRNA	Forward	5’-GGTAGACTGCCAGCCTGTTC-3’
	Reverse	5’-CAGTCACTTGCGATCTTCCA-3’
NXF1 mRNA	Forward	5’- GATGTGGCAATGAGTGATGC-3’
	Reverse	5’- TTCTTTGAGGTCCCATCCTG-3’
C9 intron1	Forward	5’- TCGCTGAGGGTGAACAAGAA-3’
	Reverse	5’-GCGCGCGACTCCTGAGT-3’
	probe	5’-FAM-AAACAACCG/ZEN/CAGCCTGTAGCAAGCTC-IBFQ-3’
C9 Exon2	Forward	5’-TGTGACAGTTGGAATGCAGTGA-3’
	Reverse	5’-GCCACTTAAAGCAATCTCTGTCTTG-3’
	probe	5’-FAM-TCGACTCTT/ZEN/TGCCCACCGCCA-IBFQ-3’

Supplementary Table 4. Primers for MiSeq library preparation.

C9ORF72 splicing reporter mRNA library round 1	Forward	5'-AGCTCTAGCAGGAAAGAG-3'
	Reverse	5'-GTGACTGGAGTTCAGACGTGTGCTCTTC CGATCTCGTCTTCGAGTGGGTAGAAT-3'
C9ORF72 splicing reporter mRNA library round 2	Forward	5'-AATGATACGGCGACCACCGAGATCTACAC TCTTTCCCTACACGACGCTCTTCCGATCTAGC TCTAGCAGGAAAGAG-3'
	Reverse	5'-CAAGCAGAAGACGGCATACGAGATNNNN NNGTGACTGGAGTTCAGACGTGTGC-3'
Endogenous *C9ORF72* mRNA library round 1	Forward	5'-GGTGTCCCGCTAGGAAAGAG-3'
	Reverse	5'-GTGACTGGAGTTCAGACGTGTGCTCTTCC GATCTGGTGATTTGCCACTTAAAGCA-3'
Endogenous *C9ORF72* mRNA library round 2	Forward	5'-AATGATACGGCGACCACCGAGATCTACAC TCTTTCCCTACACGACGCTCTTCCGATCTGGT GTCCCGCTAGGAAAGAG-3'
	Reverse	5'-CAAGCAGAAGACGGCATACGAGATNNNN NNGTGACTGGAGTTCAGACGTGTGC-3'
Circular intron round 1 (reporter and endogenous)	Forward	5'-CACATCTTTGACTTAAGAGGAC-3'
	Reverse	5'-GTGACTGGAGTTCAGACGTGTGCTCTTCC GATCTAGAGCAAGTAGTGGGGAGAG-3'
Circular intron round 2 (reporter and endogenous)	Forward	5'-AATGATACGGCGACCACCGAGATCTACAC TCTTTCCCTACACGACGCTCTTCCGATCTCAA ACCAAATATGTCTAAATCA-3'
	Reverse	5'-CAAGCAGAAGACGGCATACGAGATNNNN NNGTGACTGGAGTTCAGACGTGTGC-3'
