# Supplementary material for: Nuclear export and translation of circular repeat-containing intronic RNA in C9ORF72-ALS/FTD
Source: Nat Commun. 2021 Aug 13;12:4908. doi: 10.1038/s41467-021-25082-9 (PMC8363653; doi:10.1038/s41467-021-25082-9)
Supplement: Supplementary file 6 — Description of additional supplementary files [file 41467_2021_25082_MOESM6_ESM.docx]

Description of additional supplementary files

**Title: Supplementary Movie 1:**

Description: Diffusion of cytoplasmic introns and exons. *C9ORF72* intronic reporter (Fig. 1a) was stably expressed in U-2 OS cells. Intron was stably labeled with stdMCP-Halotag-JF646 (magenta) and exon with PCP-stdGFP (cyan). Images were acquired at 50ms exposure time for 12 seconds. Scale bar: 5 μm.

**Title: Supplementary Movie 2**:

Description: RAN translation occurs on spliced intron. *C9ORF72* translation reporter (Fig. 5a) was transiently transfected in U-2 OS cells stably expressing the membrane tethered stdMCP system. Translation sites (green, SunTag-scFv-sfGFP) were colocalized with introns (magenta, stdMCP-Halotag-CAAX-JF646) but not exons (cyan: stdPCP-stdTagRFP-T). Images were acquired with exposure time of 500 ms, in 10s (protein) and 30s (RNA) intervals for 15 minutes. Scale bar: 2 μm.

**Title: Supplementary Movie 3:**

Description: RAN translation increases with stress stimuli.*C9ORF72* translation reporter (GR in frame with Suntag) (Fig. 5a) was transiently transfected in U-2 OS cells stably expressing the membrane tethered stdMCP system. Cells were treated with 2mM sodium arsenite at time 0 and imaged every 30s for 30 minutes. Magenta (intron): stdMCP-Halotag-CAAX-JF646, green (protein): SunTag-scFv-sfGFP. Scale bar: 2 μm.

**Title: Supplementary table 5**

Description: smFISH probes used in the study
