# Supplementary material for: Nuclear export and translation of circular repeat-containing intronic RNA in C9ORF72-ALS/FTD
Source: Nat Commun. 2021 Aug 13;12:4908. doi: 10.1038/s41467-021-25082-9 (PMC8363653; doi:10.1038/s41467-021-25082-9)
Supplement: Supplementary file 7 — Reporting Summary [file 41467_2021_25082_MOESM7_ESM.pdf]

nature research

Corresponding author(s): Shuying Sun
Bin Wu

Last updated by author(s): Jul 3, 2021

Reporting Summary

Nature Research wishes to improve the reproducibility of the work that we publish. This form provides structure for consistency and transparency in reporting. For further information on Nature Research policies, see our Editorial Policies and the Editorial Policy Checklist.

Statistics

For all statistical analyses, confirm that the following items are present in the figure legend, table legend, main text, or Methods section.

n/a	Confirmed	
☐	☒	The exact sample size (n) for each experimental group/condition, given as a discrete number and unit of measurement
☐	☒	A statement on whether measurements were taken from distinct samples or whether the same sample was measured repeatedly
☐	☒	The statistical test(s) used AND whether they are one- or two-sided<br>*Only common tests should be described solely by name; describe more complex techniques in the Methods section.*
☒	☐	A description of all covariates tested
☒	☐	A description of any assumptions or corrections, such as tests of normality and adjustment for multiple comparisons
☐	☒	A full description of the statistical parameters including central tendency (e.g. means) or other basic estimates (e.g. regression coefficient) AND variation (e.g. standard deviation) or associated estimates of uncertainty (e.g. confidence intervals)
☐	☒	For null hypothesis testing, the test statistic (e.g. F, t, r) with confidence intervals, effect sizes, degrees of freedom and P value noted<br>*Give P values as exact values whenever suitable.*
☒	☐	For Bayesian analysis, information on the choice of priors and Markov chain Monte Carlo settings
☒	☐	For hierarchical and complex designs, identification of the appropriate level for tests and full reporting of outcomes
☒	☐	Estimates of effect sizes (e.g. Cohen's d, Pearson's r), indicating how they were calculated

Our web collection on statistics for biologists contains articles on many of the points above.

Software and code

Policy information about availability of computer code

Data collection	Nikon Elements v4.8 was used to collect the imaging data;<br>Bio-Rad CFX Manager v3.1 was used for qPCR data;<br>Illimina MiSeq 300nt single-end for circular intron and splicing junction sequencing in the Genetic Resources Core Facility at Johns Hopkins University;<br>MinKNOW software v 20.06.9 was used for mRNA long reads sequencing.
Data analysis	matlab R2017a and FISH-Qunta v3 were used for the analysis of single molecular FISH data;<br>CFX96 optical system software v1.1 for qPCR data analysis;<br>Minimap 2 v2.17 and Guppy v4.0.11 for sequencing data analysis;<br>Airlocalize and u-track v2.0 for live cell imaging data analysis;<br>ViennaRNA v2.4.18 for intron RNA structure analysis;<br>custom built Matlab script for intron G content analysis: https://github.com/binwulab/CircularIntron;<br>Fiji v1.48i for image visualization;<br>Graphpad prism v8.4.3 for data plotting and analysis.

For manuscripts utilizing custom algorithms or software that are central to the research but not yet described in published literature, software must be made available to editors and reviewers. We strongly encourage code deposition in a community repository (e.g. GitHub). See the Nature Research guidelines for submitting code & software for further information.

Data

Policy information about availability of data

All manuscripts must include a data availability statement. This statement should provide the following information, where applicable:

- Accession codes, unique identifiers, or web links for publicly available datasets
- A list of figures that have associated raw data
- A description of any restrictions on data availability

All sequencing data are available and can be accessed: https://www.ncbi.nlm.nih.gov/Traces/study/?acc=PRJNA660882. Source data are provided with this paper.

Field-specific reporting

Please select the one below that is the best fit for your research. If you are not sure, read the appropriate sections before making your selection.

☒ Life sciences ☐ Behavioural & social sciences ☐ Ecological, evolutionary & environmental sciences

For a reference copy of the document with all sections, see nature.com/documents/nr-reporting-summary-flat.pdf

Life sciences study design

All studies must disclose on these points even when the disclosure is negative.

Sample size	No sample size calculation was performed. All experiments were performed using sample size based on standard protocols in the field. Unless otherwise noted, n=3 biological replicates were used.
Data exclusions	No data were excluded
Replication	All experiments were repeated a minimum of three times. We specified the number of biological replicates in the respective figure legends. All replication attempts were successful.
Randomization	We plated the cell in random positions in multi-well plates and randomly assigned them to different experiment groups. We randomly took fluorescence images under microscope.
Blinding	The researchers who performed nanopore sequencing were blinded to the genotype. Blinding was not relevant to mi-seq as sequencing libraries were mixed before loading. Technician who performed the ELISA is blinded to the genotype and treatment. Other data collection and analysis were not performed blind to the condition. The same results have been repeated by multiple members of the research team.

Reporting for specific materials, systems and methods

We require information from authors about some types of materials, experimental systems and methods used in many studies. Here, indicate whether each material, system or method listed is relevant to your study. If you are not sure if a list item applies to your research, read the appropriate section before selecting a response.

Materials & experimental systems

n/a	Involved in the study	
☐	☒	Antibodies
☐	☒	Eukaryotic cell lines
☒	☐	Palaeontology and archaeology
☒	☐	Animals and other organisms
☒	☐	Human research participants
☒	☐	Clinical data
☒	☐	Dual use research of concern

Methods

n/a	Involved in the study	
☒	☐	ChIP-seq
☒	☐	Flow cytometry
☒	☐	MRI-based neuroimaging

Antibodies

Antibodies used	Immunoblotting:<br>anti-NXF1 (1:1000, Bethyl Laboratories, #A300-914A); anti-β-actin (1:1000, Cell Signaling Technology, #3700); Donkey anti-rabbit IgG HRP conjugated (1:5000, GE healthcare, #NA934V); Sheep anti-mouse IgG HRP conjugated (1:5000, GE healthcare, #NA931V).<br>Immunofluorescence:<br>anti-sfGFP (1:5000, Aves Labs, GFP-1010); Goat anti-Chicken IgY (H+L) Secondary Antibody, Alexa Fluor 647 (1:1000, Thermo Fisher Scientific, A21449).
Validation	The antibodies used in this work are all commercial and have been validated. Detailed information can be found on the website from the manufacturer or related publications listed below.

NXF1 (A300-914A): https://www.bethyl.com/product/A303-914A/NXF1+Antibody#
β-actin (3700): https://www.cellsignal.com/products/primary-antibodies/b-actin-8h10d10-mouse-mab/3700
Donkey anti-rabbit IgG HRP conjugated (NA934V): https://www.bioz.com/result/donkey%20anti%20rabbit%20na934v/product/GE%20Healthcare
Sheep anti-mouse IgG HRP conjugated (NA931V): https://www.bioz.com/result/NA931V/product/GE%20Healthcare
sfGFP(GFP-1010: https://www.aveslabs.com/products/anti-green-fluorescent-protein-antibody-gfp?variant=25144111169636;
Goat anti-Chicken IgY (H+L) Secondary Antibody, Alexa Fluor 647(A21449)https://www.thermofisher.com/antibody/product/Goat-anti-Chicken-IgY-H-L-Secondary-Antibody-Polyclonal/A-21449

Eukaryotic cell lines

Policy information about cell lines

Cell line source(s)	Patient and control fibroblasts were gifts from Dr. John Ravits.<br>Patient lymphoblast cells were obtained from Coriell Institute. And the catalog numbers are listed in supplementary table 1.<br>U-2 OS (American Type Culture Collection HTB-96)<br>HEK293T (American Type Culture Collection CRL-1573
Authentication	We have done authentication for U2OS cells. We have done repeat prime PCR to confirm the (GGGGCC)n expansion in patient fibroblast and lymphoblast cells. All the lines have been previously published.
Mycoplasma contamination	All cell lines were mycoplasma negative as tested in house using the PlasmoTest™ - Mycoplasma Detection Kit (InvivoGen).
Commonly misidentified lines<br>(See ICLAC register)	No commonly misidentified lines were used
